# Diagnostic yield of dental radiography and digital tomosynthesis for the identification of anatomic structures in cats

**DOI:** 10.3389/fvets.2024.1408807

**Published:** 2024-05-02

**Authors:** Maria M. Soltero-Rivera, Richard Nguyen, Stephanie Lynne Goldschmidt, David C. Hatcher, Boaz Arzi

**Affiliations:** School of Veterinary Medicine, Veterinary Surgical and Radiological Sciences, University of California, Davis, Davis, CA, United States

**Keywords:** oral anatomy, cats, digital tomosynthesis, dentition, dental radiography, imaging

## Abstract

**Introduction:**

Digital tomosynthesis (DT) has emerged as a potential imaging modality for evaluating anatomic structures in veterinary medicine. This study aims to validate the diagnostic yield of DT in identifying predefined anatomic structures in feline cadaver heads, comparing it with conventional intraoral dental radiography (DR).

**Methods:**

A total of 16 feline cadaver heads were utilized to evaluate 19 predefined clinically relevant anatomic structures using both DR and DT. A semi-quantitative scoring system was employed to characterize the ability of each imaging method to identify these structures.

**Results:**

DT demonstrated a significantly higher diagnostic yield compared to DR for all evaluated anatomic structures. Orthogonal DT imaging identified 13 additional anatomic landmarks compared to a standard 10-view feline set obtained via DR. Moreover, DT achieved statistically significant higher scores for each of these landmarks, indicating improved visualization over DR.

**Discussion:**

These findings validate the utility of DT technology in reliably identifying clinically relevant anatomic structures in the cat skull. This validation serves as a foundation for further exploration of DT imaging in detecting dentoalveolar and other maxillofacial bony lesions and pathologies in cats.

## Introduction

Dental radiography (DR) is the current standard of practice for obtaining diagnostic images of the dentoalveolar structures in dogs and cats (1, 2). Full mouth DR in cats involves 10 standard projections (2) that allow the clinician to evaluate critical anatomy such as the structural integrity of the teeth, periodontal ligament, and alveolar bone to determine the extent of periodontal or endodontal disease, presence of feline resorptive lesions, and other aberrant pathology (1, 2). However, traditional radiography is limited by its two-dimensional nature (2D) of three-dimensional (3D) structures that are prone to superimposition artifacts leading to inability to evaluate all aspects of any structure individually. As such, DR interpretation requires special considerations of the superimposition. Adequate positioning and technique are required to obtain DR images that are representative and diagnostic. A diagnostic quality DR image set should also be performed in a timely manner which may be technically challenging and requires training and a has a high and steep learning curve. Consequently, veterinary medicine can benefit from alternative imaging methods that are less technique sensitive and that eliminate superimposition of structures thereby facilitating interpretation of images.

**Figure 1:**
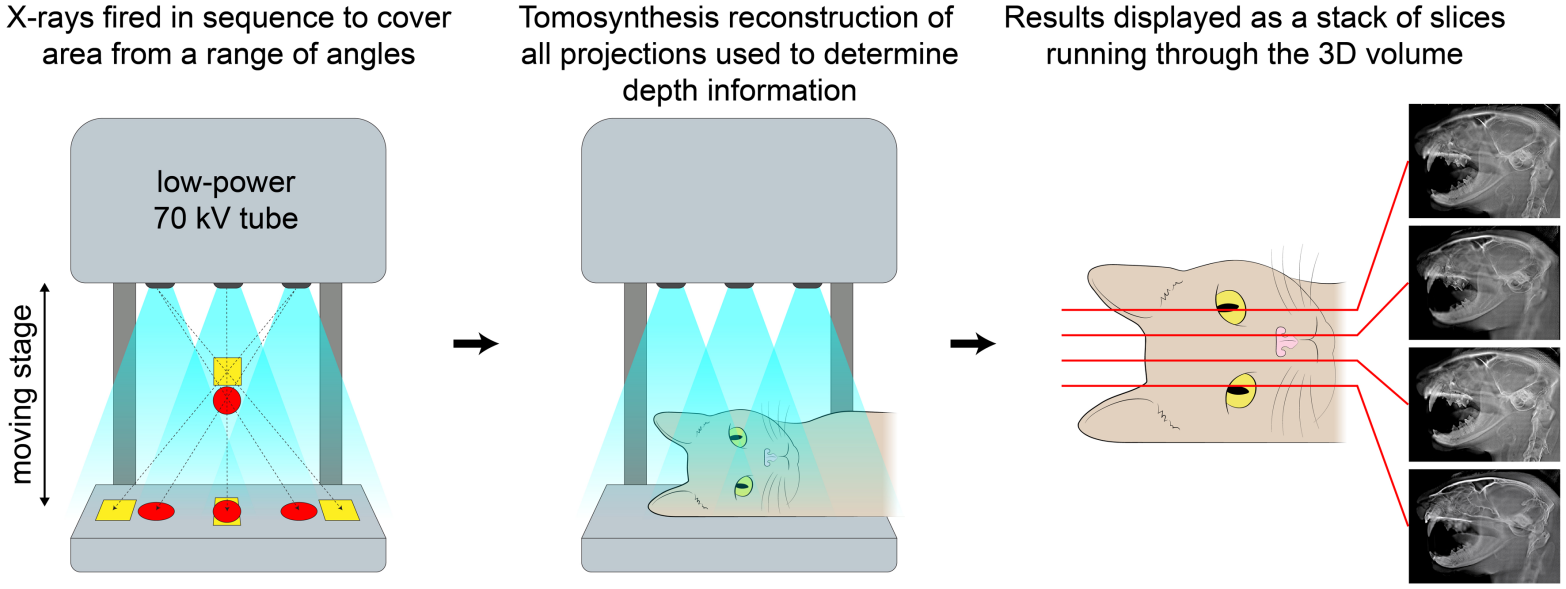
Schematic showing how images are obtained, reconstructed and displayed in digital tomosynthesis.

In human medicine, digital tomosynthesis (DT) has been proven as an important imaging modality for obtaining information that retains 3D spatial resolution without being affected by superimposition. DT creates pseudo-3D image series by obtaining multiple 2D radiographs at different angles to generate cross-sectional images that are compiled together for interpretation (Figure 1). The image series can pan through each slice, moving through the volume of the imaged subject for evaluation, in a similar manner as with computed tomography (CT) while resulting in a lower radiation dose (3). However, when comparing the dose of DT with cone beam CT (CBCT) for imaging of the paranasal sinuses, the effective dose for the CBCT and DT examinations were 30 μSv and 65 μSv, respectively, (4). Mammograms performed via DT are the current standard for screening breast cancer, and its application in screening for pulmonary nodules provides a valuable alternative to full thoracic CT with better diagnostic yield compared to conventional radiography in human medicine (5, 6).

Most relevantly, DT has been explored for use in human dentistry with positive results. In one study, anthropomorphic phantoms, incorporating teeth featuring simulated and authentic caries lesions, underwent imaging with a dose comparable to D-speed film dose. Tomosynthesis images of the phantom and teeth specimen exhibited perceived image quality equal to or surpassing standard digital images, along with the advantage of 3D information (7). Another study showed DT was able to visualize more anatomical regions. DT achieved visualization of smaller structures without superimposition, elimination of metal artifacts and higher spatial resolution as compared to CT was also noted (8).

In veterinary dentistry and oral surgery, there has been no standardized applications of DT to date. Advantages of DT over DR include faster imaging acquisition, more leniency in patient positioning, and ability to evaluate 3D information without superimposition. In addition, DT may be less technique sensitive and thus more reliable in obtaining images that are of diagnostic quality and reproduceable. However, this assumption has not been validated to ensure that the quality of information obtained is at least comparable to that of DR (2, 8).

The objective of this study was to evaluate the diagnostic yield of DT imaging as compared to standard intraoral DR for evaluating clinically relevant anatomical landmarks in the cat’s skull. Nineteen predefined anatomic structures were evaluated using both imaging modalities, and the images were interpreted using a semi-quantitative system. We hypothesized that DT would outperform DR in identifying clinically relevant anatomic structures of the feline oral cavity.

## Materials and methods

### Animals

Sixteen feline cadaver non-brachycephalic heads of unknown breed and sex were evaluated. These cats were euthanized for reasons unrelated to this study. Once obtained, the heads were screened by two board-certified dentists (MSR and BA) for grossly obvious periodontal or endodontal disease as well as congenital or acquired maxillofacial pathology that would impact anatomic quality. All cats were determined free of observable pathosis.

### Image acquisition

Dental radiography (DR) and digital tomosynthesis (DT) were performed on the cadaver heads.

Full mouth DR images were obtained using a digital intraoral imaging system (Heliodent MD, Siemens Sirona; ScanX, Air Techniques) at 60 kVp, 7 mA, and exposure time of 0.12–0.20 s (depending on the location of the evaluated structures). This system yielded a resolution of up to 18 linepairs/mm, which equated to a pixel size of 55.5 μm. Radiographic images included the standard series of views in accordance with American Veterinary Dental College guidelines (1, 2).

A DT unit (Adaptix Flat-Panel X-ray Source) was used to obtain the second image series. Serial slices of the cadaver head were obtained such that the total height of the skull (rounded up to the nearest 10 mm) was divided into 50 evenly spaced slices to create the DT image series. The DT system has a pixel size of 99 μm, which yields a resolution of up to 5 linepairs/mm. Guidelines to explain the ideal technique for obtaining DT images to optimize the visualization of landmarks do not exist; thus, image optimization was performed during image acquisition. For the lateral view, the heads were placed on the right lateral recumbency and maintained with open mouth during the scan with a 23 mm length mouth prop created using a 1 mL syringe (Vetrijec TB syringe, Vet One), inner diameter 5 mm, placed on the left maxillary and mandibular canine teeth. The crosshair was centered over the temporomandibular joint on lateral views. The view obtained in lateral recumbency allowed for the evaluation of all landmarks, including teeth. However, the teeth presentation in each view varied from right to left unless the nose was propped up to allow for the TMJs to be centered over each other. The resulting images would not show all the teeth in the same plane, similar to the images produced by DR; thus, panning was necessary to assess each tooth on a sagittal plane.

Dorsoventral views were also obtained using a mouth prop to increase the distance between the maxillary and mandibular teeth. The crosshair for the dorsoventral view was aligned sagittal over the midline and centered at the rostral zygoma. This view allowed for the evaluation of anatomical landmarks on a coronal plane. A single investigator (RN) obtained the DR and DT images, with optimization and input from a board-certified veterinary dentist (MSR).

### Image evaluation and scoring

**Figure 2:**
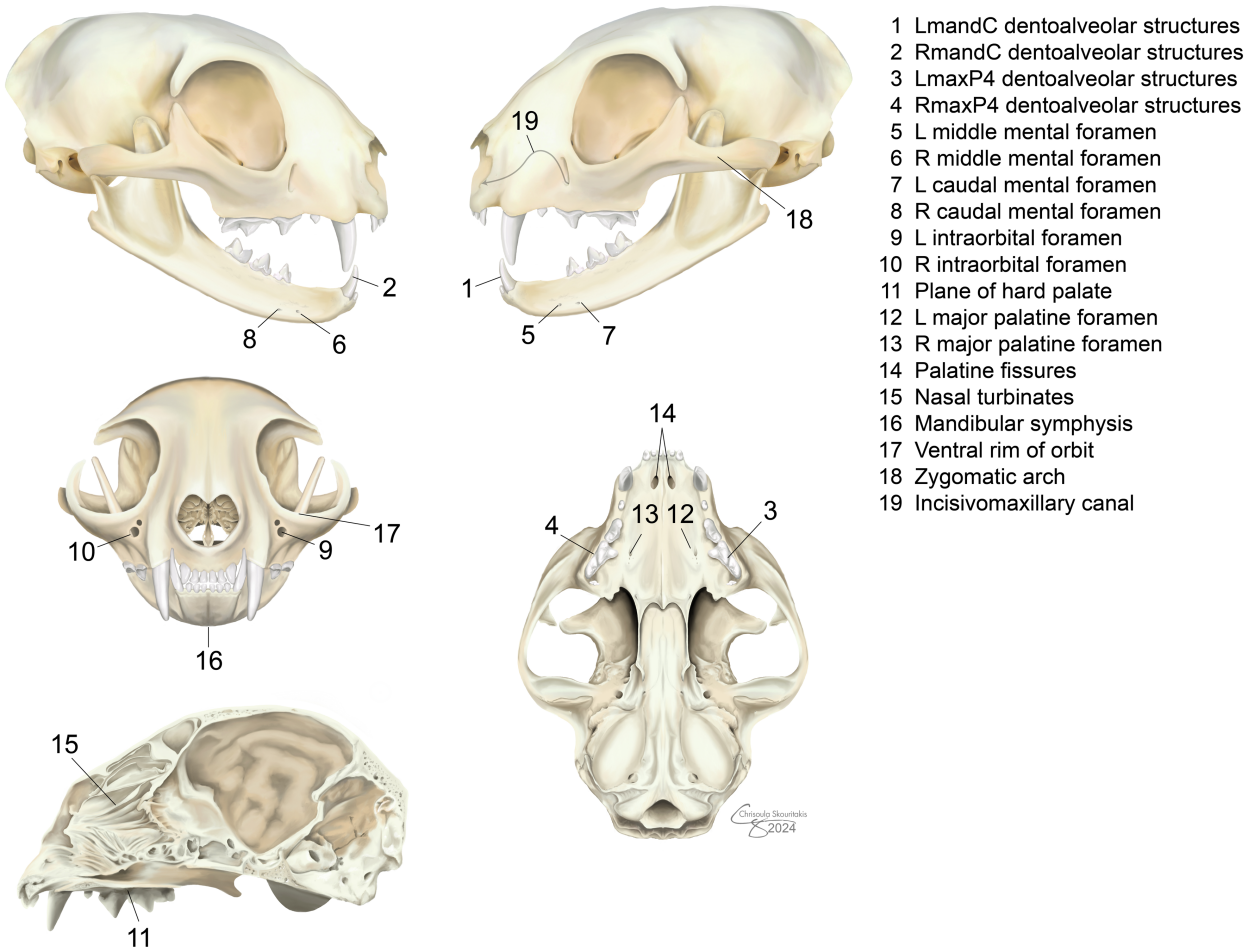
Predefined anatomic structures evaluated in cats using dental radiography and digital tomosynthesis.

The DR and DT were evaluated separately for the ability to identify 19 predefined clinically relevant anatomic structures (Figure 2) and the quality of identification. Dentoalveolar structures refers to the radiological identification of anatomical components of the oral cavity that are directly related to the teeth and their supporting tissues within the alveolar bone including: teeth, alveolar bone, and periodontal ligament space. The DR images were uploaded to a confidential repository, and files were randomized for remote, blinded evaluation using the same software (Progeny Imaging Software, Midmark). The DT images were stored on an online, cloud-based, secure image viewer (Cimar, Cimar UK Ltd). Two board-certified veterinary dentists evaluated both image types (BA, SG). Raters evaluated images separate from each other and were instructed to start with DT images first.

A semi-quantitative scoring system was used for each imaging method. Scoring for DT images was performed after evaluation of both views. Scoring was on a scale of 0–1 for the presence of the structure (0 = unable to identify, 1 = able to identify), and on a scale of 0–3 for quality of identification (0 = unable to identify, 1 = poor visualization, 2 = fair identification, 3 = excellent identification). Using each imaging method, the mean scores over the 16 cadaver heads were calculated for each anatomic structure. A total mean score of the imaging method for evaluating all 19 anatomic structures was calculated and reported as poor (mean score < 1), moderate (mean score ≥ 1 and < 2), fair (mean score ≥ 2 and < 3), and excellent (mean score = 3) (9).

### Dental radiography

The software showed images on the labial mount. Contralateral images were simultaneously evaluated for each view to allow for comparison, and software tools such as zoom and contrast adjustment were used to optimize visualization of the structure of interest.

### Digital tomosynthesis

A single investigator (RN) selected two key images per series per view (ex. 2 key images for the DV, 2 for the R lateral) that served as starting points for panning through the DT image series for each cat skull in the software. Panning started on the right side for the lateral study and the ventral aspect for the DV study. Contrast levels and magnification were manually adjusted as needed to optimize the evaluation of each anatomic structure.

### Statistical analysis

Descriptive statistics and mean scores were reported as mean ± SD. Scores from all patients for each anatomical structure and each imaging method were used to calculate the overall mean ± SD. The Wilcoxon signed-rank test was used to test for differences in scores for each anatomical region for the two imaging modalities. The score for each region and image is the average of the two raters. Significance was set at values of *p* < 0.05. Kappa coefficient for inter-rater agreement could not be assessed due to sample size and occurrence of perfect agreement between raters. Instead, the percentage of occurrence of the same score for raters per landmark and specimen was calculated for each imaging method.

## Results

### Animals

No information regarding sex, breed or age were provided for the 16 feline cadaver heads used. However, all heads had the characteristic appearance of domestic shorthaired cats. Based on pulp cavity size seen on the obtained images, the 16 cadaver heads were approximated to be consistent with 5 juvenile (< 12 months), 5 young adult (12–32 months), and 6 mature adult cats (> 32 months) (10).

### DR method

Table 1 displays which dental radiographic views were utilized to evaluate each of the 19 anatomical landmark marks.

**Table 1:** Anatomical landmarks evaluated in the study and corresponding dental radiographic (DR) views used to assess each of these, bilaterally.

Anatomical landmark	DR view(s)
Mandibular canine dentoalveolar structures	Mandibular incisor teeth; occlusal Mandibular canine teeth; lateral
Maxillary fourth premolar dentoalveolar structures	Maxillary premolar/molar teeth; lateral
Middle mental foramen	Mandibular incisor teeth; occlusal Mandibular canine teeth; lateral
Infraorbital foramen	Maxillary incisor teeth; occlusal Maxillary premolar/molar teeth; lateral
Plane of the hard palate	Maxillary canine teeth; lateral Maxillary premolar/molar teeth; lateral
Major palatine foramen	Maxillary premolar/molar teeth; lateral
Palatine fissures	Maxillary incisor teeth; occlusal
Nasal turbinates	Maxillary incisor teeth; occlusal
Mandibular symphysis	Mandibular incisor teeth; occlusal
Ventral rim of orbit	Maxillary premolar/molar teeth; lateral Maxillary premolar/molar teeth; lateral
Zygomatic arch	Maxillary premolar/molar teeth; lateral Maxillary premolar/molar teeth; lateral
Incisivomaxillary canal	Maxillary incisor teeth; occlusal Maxillary canine teeth; lateral

### DT method

In the absence of guidelines for the evaluation of DT images both the lateral and dorsoventral views were used to evaluate the 19 anatomical landmark marks. Table 2 shows which views resulted in better visualization of each landmark.

**Table 2:** Anatomical landmarks evaluated in the study and corresponding digital tomosynthesis (DT) views used to assess each of these, bilaterally.

Anatomical landmark	DT view(s)
Mandibular canine dentoalveolar structures	Dorsoventral and lateral
Maxillary fourth premolar dentoalveolar structures	Dorsoventral and lateral
Middle mental foramen	Lateral
Infraorbital foramen	Dorsoventral and lateral
Plane of hard palate	Lateral
Major palatine foramen	Dorsoventral
Palatine fissures	Dorsoventral
Nasal turbinates	Dorsoventral and lateral
Mandibular symphysis	Dorsoventral
Ventral rim of orbit	Lateral
Zygomatic arch	Dorsoventral and lateral
Incisivomaxillary canal	Lateral

### Overall scores

**Figure 3:**
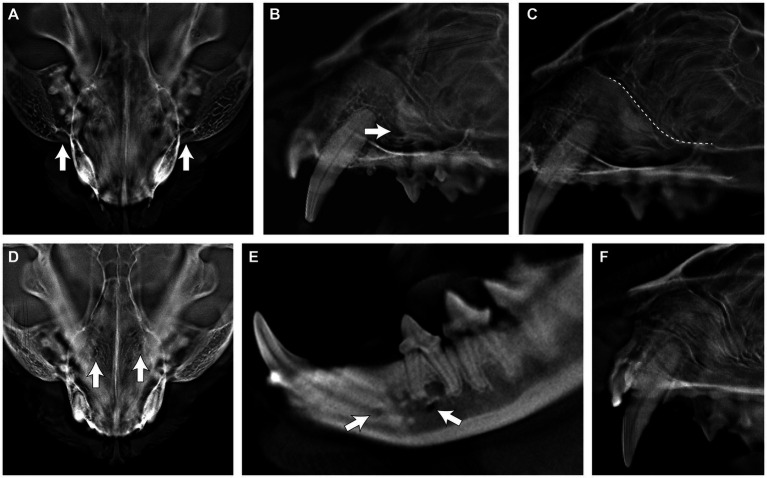
Appearance of the infraorbital foramen **(A,B)**, ventral rim of the orbit (C), major palatine foramen (D), middle and caudal mental foranima (E), and nasal turbinates (F) on digital tomosynthesis (DT).

The following structures could not be reliably identified on DR obtaining scores of < or = 1 (i.e., poor to moderate): infraorbital foramen, middle and caudal mental foramina, ventral rim of the orbit, incisivomaxillary canal, major palatine foramen, and nasal turbinates. However, all structures were identified in DT with scores of >1 to 3 (i.e., moderate to excellent). Figure 3 shows examples of the anatomical landmarks that were not visualized in DR but were visualized in DT.

Kappa agreement could not be assessed due to the small sample sizes and the instances of perfect agreement between the raters. Percentage of occurrence of the same score for both raters per anatomical landmark and cadaver in each method is shown in Table 3. Perfect agreement between graders occurred for two anatomical landmarks in DR and for six anatomical landmarks in DT. Percentage agreement between the two raters (BA, SG) was higher in DT in all but the following landmarks: maxillary fourth premolar dentoalveolar structures on both sides, left middle mental foramen, left caudal mental foramen, and the major palatine foramina. As a reminder, all patients had their left side away from the detector plate.

**Table 3:** Percentage on inter-rater agreement per anatomical landmark for dental radiography (DR) and digital tomosynthesis (DT) imaging.

Anatomical landmark	Agreement on DR	Agreement on DT
Left mandibular canine dentoalveolar structures	44% (7/16)	63% (10/16)
Right mandibular canine dentoalveolar structures	50% (8/16)	63% (10/16)
Left maxillary fourth premolar dentoalveolar structures	50% (8/16)	19% (3/16)
Right maxillary fourth premolar dentoalveolar structures	63% (10/16)	19% (3/16)
Left middle mental foramen	63% (10/16)	63% (10/16)
Right middle mental foramen	31% (5/16)	63% (10/16)
Left caudal mental foramen	63% (10/16)	31% (5/16)
Right caudal mental foramen	44% (7/16)	69% (11/16)
Left infraorbital foramen	19% (3/16)	69% (11/16)
Right infraorbital foramen	25% (4/16)	69% (11/16)
Plane of hard palate	38% (6/16)	100% (16/16)
Left major palatine foramen	100% (16/16)	31% (5/16)
Right major palatine foramen	100% (16/16)	31% (5/16)
Palatine fissures	13% (2/16)	69% (11/16)
Nasal turbinates	19% (3/16)	100% (16/16)
Mandibular symphysis	38% (6/16)	100% (16/16)
Ventral rim of orbit	94% (15/16)	100% (16/16)
Zygomatic arch	63% (10/16)	100% (16/16)
Incisivomaxillary canal	94% (15/16)	100% (16/16)

**Figure 4:**
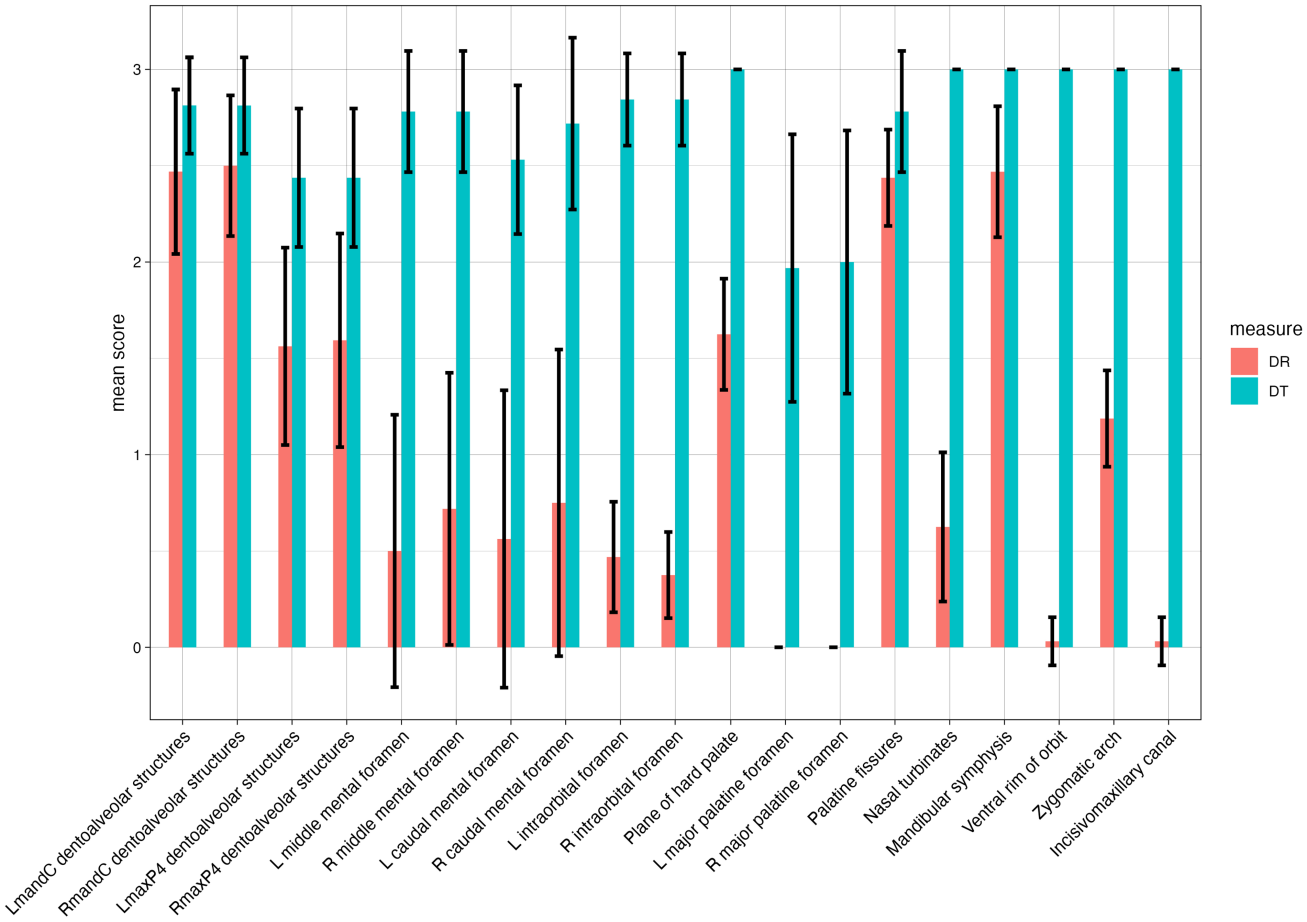
Mean combined score for both graders (SG and BA) and standard deviation for each of the 19 anatomic structures evaluated with both methods (digital tomosythesis or DT – blue; dental radiography or DR – red). A statistically significant (*p* < 0.05) difference was noted for all landmarks. Scores were assigned by use of a scale of 0–3 as follows: 0 = inability to identify the anatomic structure, 1 = poor identification of anatomic structure, 2 = good identification of anatomic structure, and 3 = excellent identification of anatomic structure.

Results from the statistical analysis for the quality of identification of the 19 landmarks for both image acquisition methods compared is depicted in Figure 4. As shown in Figure 4, DT outperformed over DR in all landmarks and this difference was statistically significant.

## Discussion

To our knowledge, this is the first study to investigate the diagnostic yield of digital tomosynthesis (DT) compared to digital dental radiography (DR) for the evaluation of dental and maxillofacial anatomic structures of cats. We demonstrate that the diagnostic yield of the DT method was superior as compared to that of the DR method. More specifically, landmarks that were not discernable in DR were visualized in DT and most had fair to excellent visualization.

Despite the innate constraints of not being able to collimate the DT machine, which may impact resolution compared to intraoral DR images, DT offered enhanced clarity and visualization of dentoalveolar structures in cats. Images acquired by the DT are digital 2-dimensional layers that are aligned perpendicular to the beam angle and stored digitally. The image quality or feature detection ability can be related to the physical characteristics of the digital images represented by sampling frequency, volume averaging, dynamic range, modulation transfer function and signal-to-noise ratio.

One of the primary advantages of DT over DR is its ability to eliminate superimposition, providing clearer and more detailed images of bone and teeth. Previous studies (11) have attempted to change the projection angle of the DR beam to reduce the superimposition of specific structures around the maxillary canine teeth on a lateral radiograph. That study found that the conchal crest, the line of conjunction between the vertical body of the maxilla and its palatine process, the incisivomaxillary canal and the lacrimal canal will superimpose on the maxillary canine tooth of mesocephalic cats (11). Though certain angulation of the radiographic beam and skull in DR may minimize superimposition it will not eliminate it completely as DT can. The angle of the radiographic beam used in our study may explain why some of these landmarks were largely missed with DR. Additionally, a study evaluating the diagnostic value of the use of lateral and occlusal radiographic views of the canine teeth in dogs revealed that lateral and occlusal radiographs of these teeth will allow assessment of all but one aspect, the palatal surface of maxillary canine teeth in dogs which is an area that the DV view on DT can show (12). Taken together, in circumstances where DR has fallen short historically, lateral and DV views obtained with DT will overperform. Examining teeth in both sagittal and coronal planes, DT offers clearer images that can enhance our understanding of the location, shape, and severity of periodontal, endodontal, and resorptive lesions in cats. This clarity may also enable earlier detection of these conditions. Moreover, DT’s improved visualization of key anatomical landmarks like the mandibular symphysis, infraorbital foramena, and nasal turbinates could contribute to the early identification and characterization of oral tumors in cats. This early detection can lead to prompt treatment and better outcomes for affected felines.

Rater agreement as assessed in this study was better in DT as compared to DR however, the statistical significance of this is unknown. Statistical assessment of inter-rater agreement presented challenges, due to small sample size and instances of perfect agreement between raters. Implementation hurdles stem from the necessity of exact or permutation tests, crucial for accurate evaluation but prone to errors in sparse cases. Additionally, common null hypothesis testing methods like Cohen’s kappa may not accurately reflect chance agreement. Consequently, inter-rater agreement was not statistically evaluated in this study. Future studies including a larger population and more raters should evaluate rater agreement via Cohen’s kappa coefficient. Of note, a higher frequency of equal scoring between raters was noted in DR for specific structures. These structures included the maxillary fourth premolar tooth which has three roots, the major palatine foramina and some left sided structures that otherwise performed well on the right side. Future studies should also evaluate the diagnostic yield of DR versus DT for the roots of multirooted teeth separately as well as examine the effect of skull width on ability to see structures bilaterally. Rater bias may also be a factor though this was minimized by performing grading blinded and separately. Additionally, DT was purposely graded first.

Overall DT time to acquisition of both the lateral and dorsoventral studies was approximately 5 min with little training and experience required to obtain diagnostic quality images consistently. Pragmatically, this translates to decreased anesthesia time for a clinical patient. In contrast, acquisition of a full DR study in a cat may take anywhere from approximately five to approximately 30 min, depending on the level of training and experience of the individual taking the radiographs. Being that DR is technique sensitive there can be inconsistency in the quality of the images obtained leading to potentially missed pathologies. This can also result in difficulties comparing studies longitudinally when individuals with different training or level of experience take the radiographs. DT may eliminate the need for intensive training and experience as well as the inconsistencies produced by DR.

Though this study highlights the higher performance of DT over DR, some considerations deserve further discussion and evaluation. Though not encountered in this study, the size of the DT receiver plate may limit its utility in larger animals. Reconstruction software in the future could provide for stitching alternatives. Additionally, effective doses from DR can range from approximately two to 170 microSv on average (13). Effective dose on cone beam computed tomography (CBCT) can vary from 19 to 1,073 microSv on average depending on the CBCT unit used and the field of view (14). Studies evaluating effective dose of a stationary-intraoral tomosynthesis imaging system are considered to be lower than conventional CT but higher than CBCT (3). Consequently, the clinical needs of the patient need to be balanced with the risk of radiation exposure. There are likely distinct effective dose variations among DT units, which can be attributed to factors including FOV, mA setting, kiloVolt (peak), scan time (including pulsed versus continuous dose), sensor sensitivity and the number of image captures. The operator can control the FOV, the mA setting and the scan time settings, which relate directly to the effective dose. Matching the FOV to the area of interest can optimize the effective dose. Having shorter scan times, reducing the mA setting or both can reduce the dose, but doing so also can decrease the signal and therefore image quality.

In conclusion, this study represents an initial investigation into the diagnostic capabilities of DT versus DR for evaluating the dentoalveolar and selected maxillofacial anatomical structures in cats. The main goal of imaging is to reveal the anatomic truth (i.e., allows for visualization and assessment of anatomy as it exists *in vivo*). The anatomic truth includes determining the spatial relationships between adjacent anatomic structures, dimensions of imaged structures and anatomic quality or health status of the imaged structures. The findings demonstrate the superiority of DT over DR, with enhanced clarity and elimination of superimposition offering a comprehensive view. While certain considerations such as the size of the DT receiver plate in larger animals, radiation exposure and potential future developments in reconstruction software warrant further discussion, the present study establishes DT as a promising alternative with a notable advantage in efficiency. Pragmatically, the shortened acquisition time, coupled with the ease of obtaining consistently high-quality images, presents a substantial reduction in anesthesia time for clinical patients. The study also addresses the limitations of DR, emphasizing the technique sensitivity that leads to potential inconsistencies and the need for extensive training. While acknowledging the benefits of DT, the potential for radiation exposure should be carefully weighed against the clinical needs of the patient. This research lays the foundation for future considerations in refining imaging techniques and establishing DT as a valuable tool in veterinary dentistry and oral surgery.

## Conclusion

In this study, we demonstrate for the first time the superior visualization capabilities of digital tomosynthesis (DT) compared to digital dental radiography (DR) in assessing the dental, alveolar and selected other maxillofacial structures in the cat. The efficient acquisition process of DT may also offer a substantial reduction in anesthesia time for clinical patients. The enhanced clarity and elimination of superimposition make DT a promising alternative for diagnosing dentoalveolar disease in cats, addressing the limitations of DR’s technique sensitivity and potential inconsistencies. While acknowledging potential limitations, this study establishes DT as a valuable advancement in veterinary dentistry, paving the way for improved diagnostic precision and patient care.

## Data availability statement

The original contributions presented in the study are included in the article/supplementary material, further inquiries can be directed to the corresponding author.

## Ethics statement

The requirement of ethical approval was waived by Institutional Animal Care and Use Committee for the studies involving animals because Cadavers donated for medical research were used in this study. No animals were euthanized for this study. The studies were conducted in accordance with the local legislation and institutional requirements.

## Author contributions

MS-R: Writing – review & editing, Writing – original draft, Supervision, Project administration, Methodology, Formal analysis, Data curation, Conceptualization. RN: Writing – review & editing, Writing – original draft, Software, Methodology, Investigation, Data curation. SG: Writing – review & editing, Methodology, Investigation, Data curation. DH: Writing – review & editing, Methodology, Formal analysis, Conceptualization. BA: Writing – review & editing, Methodology, Investigation, Data curation, Conceptualization.
